# Soy protein ingestion results in less prolonged p70S6 kinase phosphorylation compared to whey protein after resistance exercise in older men

**DOI:** 10.1186/s12970-015-0070-2

**Published:** 2015-02-05

**Authors:** Cameron J Mitchell, Paul A Della Gatta, Aaron C Petersen, David Cameron-Smith, James F Markworth

**Affiliations:** The Liggins Institute, Faculty of Medical and Science Health, University of Auckland, 85 Park Road, Grafton, Private Bag 92019, Auckland, 1023 New Zealand; Centre for Physical Activity and Nutrition Research, School of Exercise and Nutrition Sciences, Deakin University, Burwood, Melbourne Australia; Institute of Sport, Exercise and Active Living, College of Sport and Exercise Science, Victoria University, Footscray, Melbourne Australia

**Keywords:** Anabolic signalling, Supplementation, Aging, Sarcopenia

## Abstract

**Background:**

The phosphorylation of p70S6 Kinase (p70S6K) is an important step in the initiation of protein translation. p70S6K phosphorylation is enhanced with graded intakes of whey protein after resistance exercise. Soy protein ingestion results in lower muscle protein synthesis after exercise compared with whey; however, the underlying mechanisms responsible for this difference have not been reported.

**Findings:**

13 older men (60–75) completed an acute bout of lower body resistance exercise and ingested 30 g of soy protein or carbohydrate. Muscle biopsies were obtained in the rested and fasted state and 2 and 4 hours post exercise. Phosphorylation status of p70S6K was measured with western blot. Results were compared with previously reported data from the ingestion of 30 g of whey protein or placebo. p70S6K phosphorylation was increased 2, but not 4 hours post exercise with soy protein ingestion. p70S6K phosphorylation was not increased post exercise with carbohydrate ingestion.

**Conclusions:**

Ingesting 30 g of either whey or soy protein resulted in equivalent p70S6K phosphorylation at 2 hours post exercise, however, unlike whey, soy protein failed to promote prolonged phosphorylation of p70S6K to 4 hours post-exercise.

## Findings

### Background

Aging is associated with a progressive loss of muscle mass and function termed sarcopenia [1,2]. Low muscle mass and associated impaired function are emerging as important risk factors for loss of independence [3], poor survivability of many diseases [4] and ultimately all-cause mortality [5]. Reduced muscle protein synthesis (MPS) in aged muscle appears to be underpinned by deficits in the anabolic signalling response to exercise and feeding, a phenomenon which may contribute to the etiology of sarcopenia [6,7]. A better understanding of the molecular responses to exercise and nutrition in aged skeletal muscle will be important in developing effective treatment for sarcopenia.

MPS is primarily controlled by the regulation of translation initiation. The mammalian target of rapamycin (mTOR) integrates signals from nutrients, growth factors and exercise. mTOR phosphorylates downstream targets such as p70S6 kinase (p70S6K) which then phosphorylates ribosomal protein S6 (S6) [8]. In particular, phosphorylation of p70S6K has been shown to relate to both MPS and muscle hypertrophy in some but not all cases [9-13]. It is likely that p70S6K phosphorylation is a major mechanism in determining MPS and ultimately underlining long term changes in muscle mass in response to exercise and nutrition.

Previously we have shown that in agreement with measures of MPS [14], phosphorylation of p70S6K in older adults after resistance exercise responds in a dose dependant manner to increasing amounts of whey protein between 10 and 40 g [15]. In addition to protein dose, protein source and amino acid composition are known to be important determinates of the anabolic signalling response to feeding [16,17]. Yang et al. [18] have shown both at rest and after resistance exercise, that whey protein results in a greater MPS response compared to soy protein in older men. Similar findings have also been reported in young men [17], however; the underlying molecular mechanisms by which whey protein may be more effective than soy protein at stimulating MPS remain unknown. Prior work from our laboratory has shown that in response to a mixed meal enriched in either soy or dairy protein, only the dairy meal stimulated mTOR and S6 phosphorylation, whereas both dairy and soy were capable of stimulating p70S6K phosphorylation in muscle of middle aged men [19].

The mTOR pathway is also sensitive to the effects of carbohydrate via the insulin sensitive role of Akt and the energy sensing role of AMPK [8]. While it has been clearly shown that carbohydrate combined with sufficient protein does not appear to enhance MPS above that of protein alone, it is not known whether ingestion of carbohydrate would result in greater post exercise anabolic signalling response than exercise in the absence of nutrient provision [20].

The purpose of this brief communication is to characterize the time course of p70S6K phosphorylation after resistance exercise in older men in response to feeding with carbohydrate or 30 g of soy protein and contrast these data with previously published data [15] from our laboratory in which the effect of differing whey protein doses was assessed using the same experimental protocol. A secondary purpose of this study was to compare the effects of ingestion of carbohydrate alone vs a non-caloric placebo on the time course of p70S6K phosphorylation following resistance exercise.

### Methods

Thirteen healthy older men participated in this study and results were compared with those from 20 healthy older men whose characteristics have been previously reported [15]. The men were randomized to ingest either 30 g of carbohydrate (n = 6, 67.2 ± 4.7 years, 93.6 ± 15.2 kg, 179 ± 7 cm) or 30 g of soy protein (n = 7, 69.1 ± 4.5 years, 84.7.6 ± 10.4 kg, 175 ± 4 cm). This study was approved by the Deakin University Human Research Ethics Committee. The detailed exercise, sampling and analysis methods of this study have been reported previously [15]. Briefly, one week prior to the experimental session subjects completed a familiarization session and their one-repetition maximum (1RM) was estimated for the smith rack squat, leg press and leg extension exercises. Subjects arrived at the laboratory in the fasted state; a resting muscle biopsy was obtained from the vastus lateralis muscle. After a brief warmup subjects completed 3 sets of resistance exercise at 80% of their estimated 1RM for smith rack squats, leg press and leg extension in a circuit manner with 1 min rest between exercises and 3 min between sets. After the exercise bout subjects immediately ingested their randomly assigned test beverage. Further muscle biopsies were obtained at 2 and 4 hours post exercise.

Western blotting was performed to quantify the phosphorylation status of p70S6K and was expressed as fold change from rest with total ERK2 used as a loading control. Protein was separated via SDS/PAGE and then transferred to a PVDF membrane, which was incubated overnight with primary antibody against p-p70S6K (Thr389; 1:1000, Cell Signalling, Danvers, MA). After the application of a secondary antibody membranes were imaged and densitometry was performed as previously described. Original data (30 g soy and carbohydrate groups) was compared with previously published data [15] (30 g whey and placebo). Means are reported ± SEM. Differences were tested with two factor ANOVA with treatment as a between subject factor and time as a within subject factor. The Sidak post hoc method was used to compare individual groups and time points when significant interactions were present.

### Results

**Figure 1:**
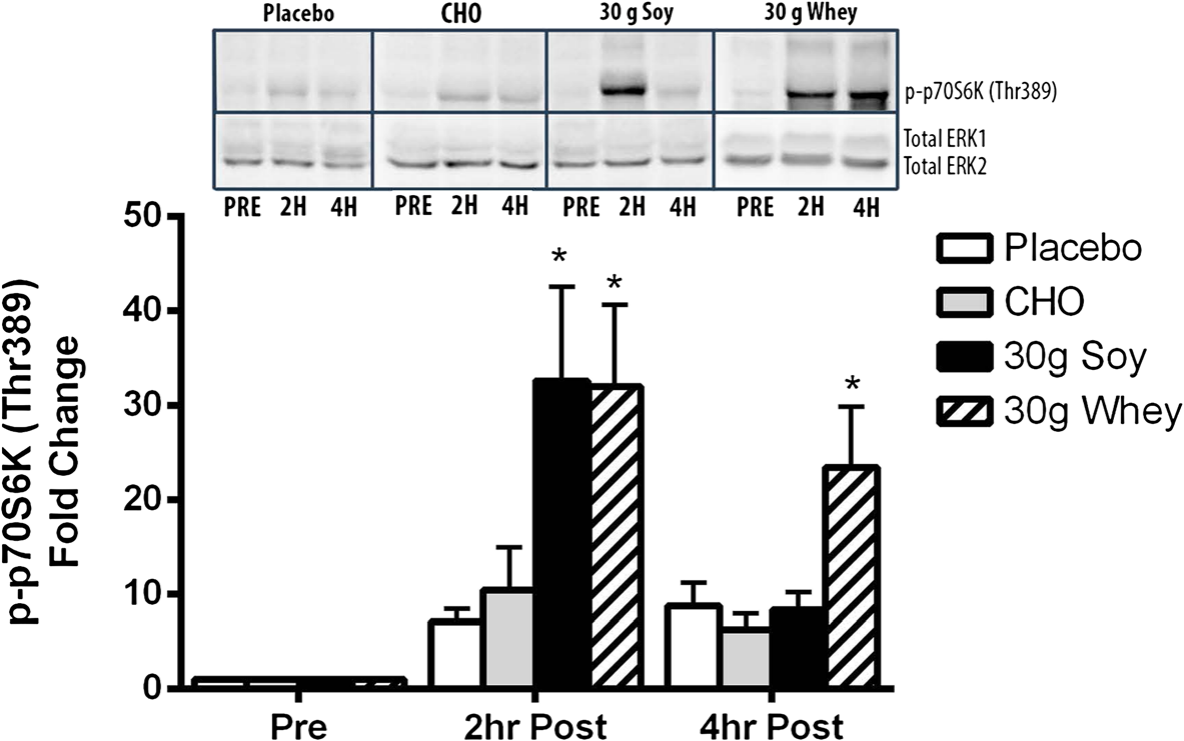
**Phosphorylation of P70S6 Kinase in response to nutritional supplements following resistance exercise in older men.** * = significantly different from pre exercise P < 0.001. The upper panel shows representative western blots for p70S6K phosphorylated at The389 and Total ERK 1&2. ERK 2 is used as a loading control. Data from the placebo (PLA) and 30 g whey group have been previously reported in reference 15.

p70S6K phosphorylation was increased above resting fasted levels at two hours post exercise in the 30 g whey and 30 g soy groups (P < 0.001), with no difference between groups. At 4 hours post exercise p70S6K phosphorylation remained elevated above baseline in the 30 g whey group (P < 0.001) but not the 30 g soy (P = 0.25) group (Figure 1). In the carbohydrate group, p70S6K phosphorylation did not significantly differ from baseline at 2 hours or 4 hours post-exercise. Placebo and whey protein dose response (10–40 g) data have been previously reported by us [15].

### Discussion

The consumption of 30 g of whey or soy protein after exercise in older men results in large increases in phosphorylation of p70S6K at 2 hours after exercise regardless of protein source [15, present study]. However, whilst whey protein resulted in elevated p70S6K phosphorylation for at least 4 hours post exercise [15], phosphorylation had returned to baseline in the group consuming a matching dose of soy protein. The physiological relevance of the time course of elevated p70S6K phosphorylation has not been firmly established however, these data fit well with MPS data from Yang et al. [18] who showed that the 4 hour average post exercise rate of MPS was elevated in older men who consumed either whey or soy protein, but was higher after the consumption of whey protein when compared to soy. Similarly over the 2.5 hour post exercise period in young men, MPS was elevated above baseline to a greater degree after the consumption of whey protein compared to soy protein [17]. Measures of MPS provide important insight into the likely phenotypic outcome of chronic post exercise protein consumption [21], however, they lack temporal resolution and do not provide insight into the underlying molecular mechanisms. Because MPS is an aggregate measure between two muscle biopsies it is likely that in the above studies the rate of MPS was not constant throughout the full measurement period. Churchward-Venne et al. [16] have shown that after resistance exercise, early (1-3 h) MPS is similar after the ingestion of whey protein or composite amino acid mixtures, however, whey protein appears to prolong both the MPS response (3-5 h) as well as the phosphorylation of p70S6K beyond that of amino acids alone.

Although not directly measured in this study it is likely that the different leucine content of whey and soy protein resulted in a greater peak concentration of leucine in the blood and a more prolonged elevation in leucine levels [17-19]. Prolonged elevation of p70S6K phosphorylation does not always translate to persistent increases in MPS due to the ‘muscle full effect’ [22], however, resistance exercise appears to extend the period of elevated MPS following feeding when p70S6K phosphorylation is also increased [16]. Our data fits well with MPS data from young and old men as well as the results of long term training studies in young men which show greater lean mass gain with post exercise whey consumption when compared with soy protein consumption [23]. Future work should establish a time course for both MPS and anabolic signalling after the consumption of whey and soy protein. Additionally, more work is required to characterize the signalling events upstream of p70S6K in order to better understand how exercise and nutritional stimuli are sensed and integrated into a signal for increased protein translation initiation and synthesis. In conclusion, this study shows that carbohydrate alone provided after resistance exercise does not augment p70S6K activation and that compared to whey [15] soy protein lead to a less prolonged p70S6K signalling response after resistance exercise in muscle of older men.

### Consent

Written informed consent was obtained from the participants for the publication of this report and any accompanying images.

## Abbreviations

1RM: One-repetition maximum
AMPK: Adenosine monophosphate-activated protein kinase
ANOVA: Analysis of variance
ERK: Extracellular signal-regulated kinases
MPS: Muscle protein synthesis
mTOR: Mammalian target of rapamycin
p70S6K: p70S6 kinase
PVDF: Polyvinyl difluoride
S6: Ribosomal protein S6
SDS/PAGE: Sodium dodecyl sulphate/polyacrylamide gel electrophoresis
SEM: Standard error of the mean
